# Seed endophytes and rhizosphere microbiome of *Imperata cylindrica*, a pioneer plant of abandoned mine lands

**DOI:** 10.3389/fmicb.2024.1415329

**Published:** 2024-07-24

**Authors:** Wenqin Mao, Ying Wu, Qiaohong Li, Yingying Xiang, Wenting Tang, Haiyan Hu, Xiuling Ji, Haiyan Li

**Affiliations:** ^1^Life Science and Technology and Medical Faculty, Kunming University of Science and Technology, Kunming, China; ^2^The First People’s Hospital of Yunnan Province, Kunming, China; ^3^The Affiliated Yanan Hospital of Kunming Medical University, Kunming, China; ^4^State Key Laboratory of Environmental Geochemistry, Institute of Geochemistry, Chinese Academy of Sciences, Guiyang, China

**Keywords:** heavy metal, Pioneer plant, *Imperata cylindrica*, seed endophyte, microbiota, revegetation

## Abstract

Some plant-associated microorganisms could improve host plants biotic and abiotic stress tolerance. *Imperata cylindrica* is a dominant pioneer plant in some abandoned mine lands with higher concentrations of heavy metal (HM). To discover the specific microbiome of *I. cylindrica* in this extreme environment and evaluate its role, the microbiome of *I. cylindrica*’s seeds and rhizosphere soils from HM heavily contaminated (H) and lightly contaminated (L) sites were studied. It was found that HM-contamination significantly reduced the richness of endophytic bacteria in seeds, but increased the abundance of resistant species, such as *Massilia* sp. and *Duganella* sp. Spearman’s rank correlation coefficient analysis showed that both *Massilia* sp. and *Duganella* sp. showed a significant positive correlation with Zn concentration, indicating that it may have a strong tolerance to Zn. A comparison of the microbiome of rhizosphere soils (RS) and adjacent bare soils (BS) of site H showed that *I. cylindrica* colonization significantly increased the diversity of fungi in rhizosphere soil and the abundance of Ascomycota associated with soil nutrient cycling. Spearman’s rank correlation coefficient analysis showed that Ascomycota was positively correlated with the total nitrogen. Combined with the fact that the total nitrogen content of RS was significantly higher than that of BS, we suppose that Ascomycota may enhance the nitrogen fixation of *I. cylindrica*, thereby promoting its growth in such an extreme environment. In conclusion, the concentration of HM and nutrient contents in the soil significantly affected the microbial community of rhizosphere soils and seeds of *I. cylindrica*, in turn, the different microbiomes further affected soil HM concentration and nutrient contents. The survival of *I. cylindrica* in HM severely contaminated environment may mainly be through recruiting more microorganisms that can enhance its nutrition supply.

## 1 Introduction

In the past 100 years, with the continuous progress and development of human society, the scale and intensity of mineral resources have increased (Gutierrez, 2020). However, during resource extraction, a large quantity of mineral waste was produced, which contains many toxic and harmful substances and poses a potential threat to the environment (Trannum et al., 2020; Xie and van Zyl, 2020). Revegetation is a plant-based technology for *in situ* restoration (Zhou et al., 2020). Vegetation restoration is environmentally sustainable and less costly than traditional physicochemical techniques (Mendez and Maier, 2008). Due to the characteristics of high heavy metal (HM) content, barren soil, and low microbial activity in the tailing area, it is generally difficult for plants to survive (De la Iglesia et al., 2006; Ginocchio et al., 2017). A previous study found that the role of the dominant plant species (*Imperata cylindrica*) was found to be important in the restoration of the plant community in the mining district (Jia et al., 2020). *I. cylindrica*, a gramineous genus, is a common plant in the wasteland and exposed tailing areas. Its rhizomes are well-developed, which can maximize the nutrients in the soil and have strong adaptability to extreme environments (Song et al., 2019). *I. cylindrica* has been shown to grow as an HMs accumulator plant in the mining district (Shaltout et al., 2016; Mahdavian et al., 2017; Li et al., 2018; Vidal et al., 2021). Previous studies showed that microbial colonization could enhance nutrient uptake and HM resistance of *I. cylindrica*, thereby promoting its growth in HM-stress environment (Jia et al., 2019; Liang et al., 2023).

Seeds are carriers of many endophytes. Seed endophytes are mostly passed down from generation to generation by vertical transmission, so they become the basis for the establishment of the plant endophyte community (Mao et al., 2023). Seed endophytes not only improve the viability and germination rate of seeds but also promote the growth and development of plants, thus enhancing the biotic and abiotic stress resistance of the host plant (Santoyo et al., 2016; Shahzad et al., 2016). For example, Parmar et al. (2022) found that seed endophyte *Epicoccum nigrum* (FZT214) can directly affect the reclaimed plants by utilizing hormone regulation and antioxidant stress, to alleviate HM stress in the mine environment and promote plant growth and “site fitness.” In addition, when the pioneer plant seeds migrated to the exposed tailing area, the endophytic bacteria could provide some nutrients for the pioneer plant by producing plant hormones, phosphorus solubilizing, potassium solubilizing, and nitrogen-fixing (Islam et al., 2021). Wang et al. (2020) and Mahmud et al. (2021) found that *Epichloë* can give plants stronger environmental tolerance by adjusting soil physical and chemical properties and changing the soil microbiome. Therefore, in the process of vegetation reclamation in the exposed tailing area, seed endophytes can significantly promote seed colonization, germination, and seedling growth of pioneer plants, enhance their heavy metal resistance, and facilitate vegetation reclamation in the tailing area (Ultra and Manyiwa, 2021).

Soil microorganisms can change the soil pH value, the physical, and chemical properties, and soil respiration rate, etc. by producing various active ingredients and play an extremely important role in soil nutrient cycling, organic matter content, biogeochemical cycling, and plant biomass (Zhao et al., 2021; Naz et al., 2022). Zhao et al. (2021) found that different vegetation reclamation modes in mining areas have different soil microbial communities, which in turn feed on the development of the plant community. Gazitúa et al. (2021) confirmed the important role of the soil microbiome in mining adaptability, early colonization, and pioneer plant growth. Therefore, soil biomes play a key role in the restoration of degraded terrestrial ecosystems (Dangi et al., 2012; Cheng et al., 2022).

However, the role of microorganisms on pioneer plants’ survival in the tailing area with higher concentrations of HM is still unknown. It was supposed that the pioneer plant growing in HM heavily contaminated sites may contain some special seed endophytes, which benefited them to grow in these extreme environments. And the colonization of these pioneer plants may further change the rhizosphere microbial community, which benefit the other plants growth by colonization of these special endophytes in heavy metals contaminated areas. Therefore, in the present study, culture-independent technology was used to analyze the microbial communities of seeds and rhizosphere soils of *I. cylindrica*, a pioneer plant of an abandoned Pb-Zn tailing area.

## 2 Methods

### 2.1 Study sites, seed and soil sampling

The study site located in Puxiong town, Jianshui county, Yunnan province, Southwest China. One sampling site is the Pb-Zn tailing area (site H-HM heavily contaminated) (23°30′26″ N, 103°1′13″ E), with an altitude of 1924.9 m, and the vegetation was sparse, *I. cylindrica* was a dominant pioneer plant there. The other sampling site L (site L-HM lightly contaminated) is 5 km away from site H (23°30′21″ N, 103°2′18″ E), with an altitude of 1896.5 m. Soil contents of total potassium (TK) and total phosphorus (TP) were significantly lower at L site than at H site. And the concentrations of Pb, Zn, and Cd at L site were significantly lower than those at H site (*p* < 0.05, *t*-test) (Table 1). The sample was collected on November 25, 2020.

**Table 1:** The chemical properties of soils from two sites (mean ± SD).

Sample	TN (mg/kg)	TP (mg/kg)	TK (mg/kg)	Pb (mg/kg)	Zn (mg/kg)	Cd (mg/kg)	pH
H (BS)	381 ± 19.67	545.33 ± 6.69	11521.33 ± 718.14	3119.33 ± 201	1360.67 ± 40.08	2.89 ± 0.06	6.65 ± 0.11
RS	523 ± 18.23*	432 ± 18.58*	5266.67 ± 693.15*	1869.67 ± 167.49*	1083 ± 41.9*	2.83 ± 0.28	7.12 ± 0.02*
L	974.67 ± 34.28*	408.33 ± 3.38*	17341 ± 457.2*	123 ± 6.24*	161 ± 4.04*	0.66 ± 0.26*	6.86 ± 0.04

“*” indicates that the value is significantly different from the mean of site H (BS) (*p < 0.05, *t*-test), means ± SD (*n* = 3).

TN, Total Nitrogen; TP, Total Phosphorus; TK, Total Potassium. H, Heavy metal heavily contaminated site; L, Heavy metal lightly contaminated site; BS, Bare soils of site H; RS, Rhizosphere soils of site H.

The S-type sampling method was used to randomly select 15 healthy plants from sites H and L respectively, about 40 m^2^ area of each site were chosen for sampling and the rhizosphere soil was collected by shaking root method (Teixeira et al., 2010). Simultaneously, the background soil (without plants growing) at a depth of 5–10 cm adjacent to the sampling plants was collected. Each sample was placed separately into a sterile plastic bag, labeled, and transported to the laboratory, and the seeds were surface sterilized within 24 h. *I. cylindrica* seeds from site H and L were mixed, respectively, and evenly divided into three portions, and 0.25 ± 0.023 g was taken from each portion. Then, the seeds were surface sterilized by immersing in 75% (v/v) ethanol for 2.5 min, and were extensively rinsed with sterile distilled water five times, followed by repeating the above procedures one time (Li et al., 2012). The efficacy of the surface sterilization was checked by following the imprint method. Meanwhile, 20 ± 0.75 g soils were taken from BS and RS of site H. Thereafter, the samples were homogenized in liquid nitrogen. The seeds and soil samplings were then stored to −80°C, respectively.

### 2.2 Soil properties

The soils were ground using a high-speed blender, and the content of Pb, Zn, total nitrogen, total phosphorus, and total potassium were determined by Inductively Coupled Plasma-Atomic Emission Spectrometry (ICP-AES) (Li et al., 2016), and the concentration of Cd was analyzed by Inductively Coupled Plasma-Mass Spectrometry (ICP-MS) (Chen et al., 2022).

### 2.3 The microbiome of seed endophytes and soils

#### 2.3.1 DNA extraction and PCR amplification

The total genomic DNA was extracted taking approximately 0.2 mg of homogenized powdered samples using the MoBio PowerSoil® DNA Isolation Kit (MO BIO Laboratories, Inc., Carlsbad, CA, United States) following the manufacturer’s protocol. Extracted DNA wasverified by electrophoresis on a 1.5% (w/v) agarose gel. The qualified DNA samples were stored at −20°C for subsequent analyses.

Amplification of the seed endophytic bacterial 16S rRNA V3-V4 region was performed using primer 799F (5’-AACMGGATTA GATACCCKG-3′) and 1193R (5’-ACGTCATCCCCACCTTCC-3′) resulting in amplicons of approximately 394 bp. And amplification of the soil bacterial 16S rRNA V3-V4 region was performed using primer 338F (5’-ACTCCTACGGGAGGCAGCAG-3′) and 806R (5’-GGACTACH VGGGTWTCTAAT-3′) resulting in amplicons of approximately 468 bp. In addition, amplification of the fungal 18S ITS1 (internal transcribed spacer 1) region was performed using primer ITS1-F (5’-CTTGGTCA TTTAGAGGAAGTAA-3′) and ITS2-R (5’-GCTGCGTTCTTCAT CGATGC-3′) resulting in amplicons of approximately 350 bp. PCR reactions were performed in a 25 μL volume and contained: 2.5 μL 10× PCR buffer, 1.5 μL Mg^2+^ (25 mM MgCl_2_), 2.5 μL dNTP mixture (4 mM each), 0.5 μL KOD-PlusNeo (1 units μL^−1^; TOYOBO), 1 μL Template DNA (0.4 ng), 2.5 μL primer (10 μM each) and 14.5 μL sterilized double-distilled H_2_O. The PCR program consisted of an initial denaturation step at 94°C for 5 min, followed by 30 cycles of denaturation at 94°C for 20 s, annealing at 50°C for 30 s, elongation at 72°C for 30 s, with a final extension of 5 min at 72°C. The PCR products were purified with an OMEGA Gel Extraction Kit (Omega Bio-Tek, United States) according to the manufacturer’s protocol. The resulting amplicons were subsequently subjected to high-throughput sequencing using the Illumina MiSeq platform (Illumina, 2013). All steps were implemented at Shanghai Majorbio Bio-pharm Technology Company (Shanghai, China). The Illumina sequencing data are available in the NCBI Sequence Read Archive (SRA) repository with the BioProject accession number PRJNA1037316.

#### 2.3.2 Analysis of sequence data

The raw Illumina MiSeq sequencing data were obtained in FASTA files along with sequencing quality files. Paired-end reads from the original DNA fragments theoretically were merged using FLASH v.1.2.11, and files were accessed using MOTHUR v.1.30.2 bioinformatics software for further processing and analyses. All sequences were denoised before barcodes, and primers were removed. The cleaned-up sequences were aligned and classified along known sequences in the SILVA v.138 rRNA database. Next, chimeric sequences were detected using the UCHIME algorithm, and the remaining sequences were assigned to OTUs based on a 97% similarity criterion. Rarefaction curves were performed to check the sample adequacy using a 50 sequence increment. Finally, taxonomic information for each OTU was used the RDP Classifier v.2.13 at 0.5 confidence threshold (Wang et al., 2007). To indicate the microbial diversity in seeds, the α-diversity indices (including Shannon’s H′ and Ace indices) were quantified in terms of OTU richness.

#### 2.3.3 Statistical analysis

Relative abundance differences among different groups were detected by Kruskal-Wallis (KW) sum-rank test. A *t*-test was used to estimate the difference of α-diversity indices of seed endophytes between sites H and L and soil microbiome between RS and BS. All statistical analyses were performed with SPSS 27.

## 3 Results

### 3.1 The effect of soil HM-contamination on seed endophyte diversity and community structure

#### 3.1.1 Seed bacterial endophytes

**Figure 1:**
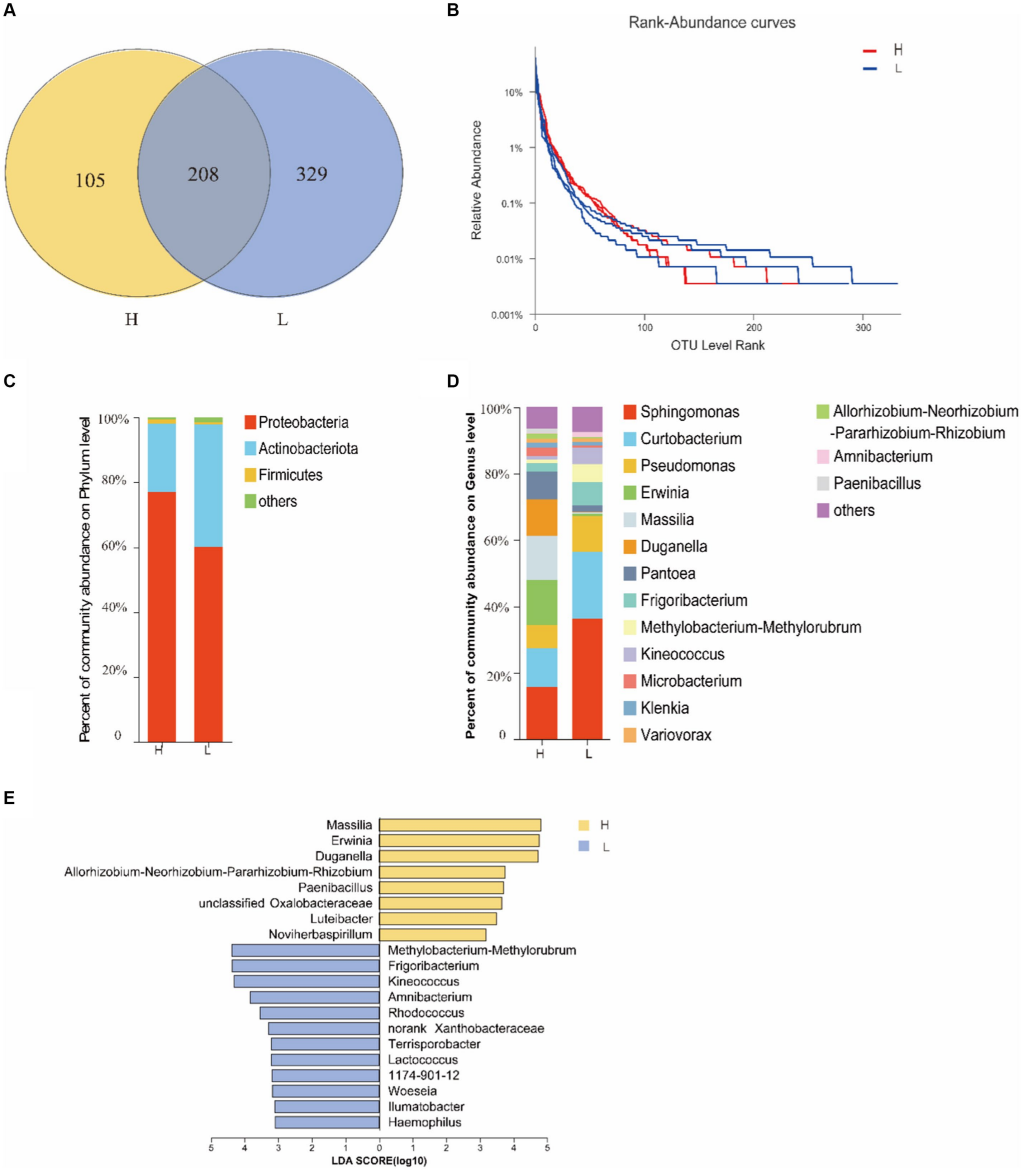
The diversity of seed bacterial endophytes of *I. cylindrica* from different heavy metal-contaminated environments. **(A)** The Venn diagram analysis of OTUs. **(B)** The rank curve based on OTU abundance. **(C,D)** Relative abundance of seed endophytic bacterial at phylum and genus level. **(E)** The indicator species of seed endophytic bacterial at genus level. H, Heavy metal heavily contaminated site; L, Heavy metal lightly contaminated site.

The OTUs of endophytic bacterial in seeds were more abundant at site L (537) than at site H (313), and 208 OTUs were shared by both two sites (Figure 1A). Rank-Abundance curve showed that the richness of seed endophytic bacteria at site L was relatively higher than that at site H (Figure 1B).

The results of 16S rRNA sequencing of seed endophytic bacterial were assigned to 19 phyla, 37 classes, 111 orders, 191 families, and 358 genera. Proteobacteria displayed the most relative abundance with sites H and L accounting for 77.01 and 60.41%, respectively (Figure 1C). At genus level, *Sphinomonas* sp. was the most dominant genus at both sites, and its relative abundance consists 36.08% at site L and 15.74% at site H. *Erwinia* sp. (13.51%) (*p* = 0.0495, Kruskal-Wallis sum-rank test) and *Massilia* sp. (13.16%) (*p* = 0.0495, Kruskal-Wallis sum-rank test) were also dominant genera at site H (Figure 1D), and their relative abundance at site H was significantly higher than that at site L (Figure 1E). Meanwhile, *Erwinia* sp. and *Massilia* sp. are also the indicator species of seed endophytic bacterial at site H at genus level (Figure 1E). It was found that the soil HM-contamination significantly decreased the richness of seed endophytic bacterial (*p* = 0.0398, *t*-test; Ace index; Table 2).

**Table 2:** The number of OTUs and α-diversity indices of seed endophytes from *Imperata cylindrica.*

Seed endophytes	Sample	Shannon’s H′	Ace
Bacteria	H	3.07 ± 0.07	247.14 ± 14.24
	L	2.71 ± 0.14	323.95 ± 17.81*
Fungi	H	2.3 ± 0.19	299.6 ± 49.29
	L	1.96 ± 0.14	263.01 ± 13.66

“*” Indicates that the value is significantly different from the mean of site H (*p < 0.05, *t*-test). H, Heavy metal heavily contaminated site; L, Heavy metal lightly contaminated site.

#### 3.1.2 Seed fungal endophytes

**Figure 2:**
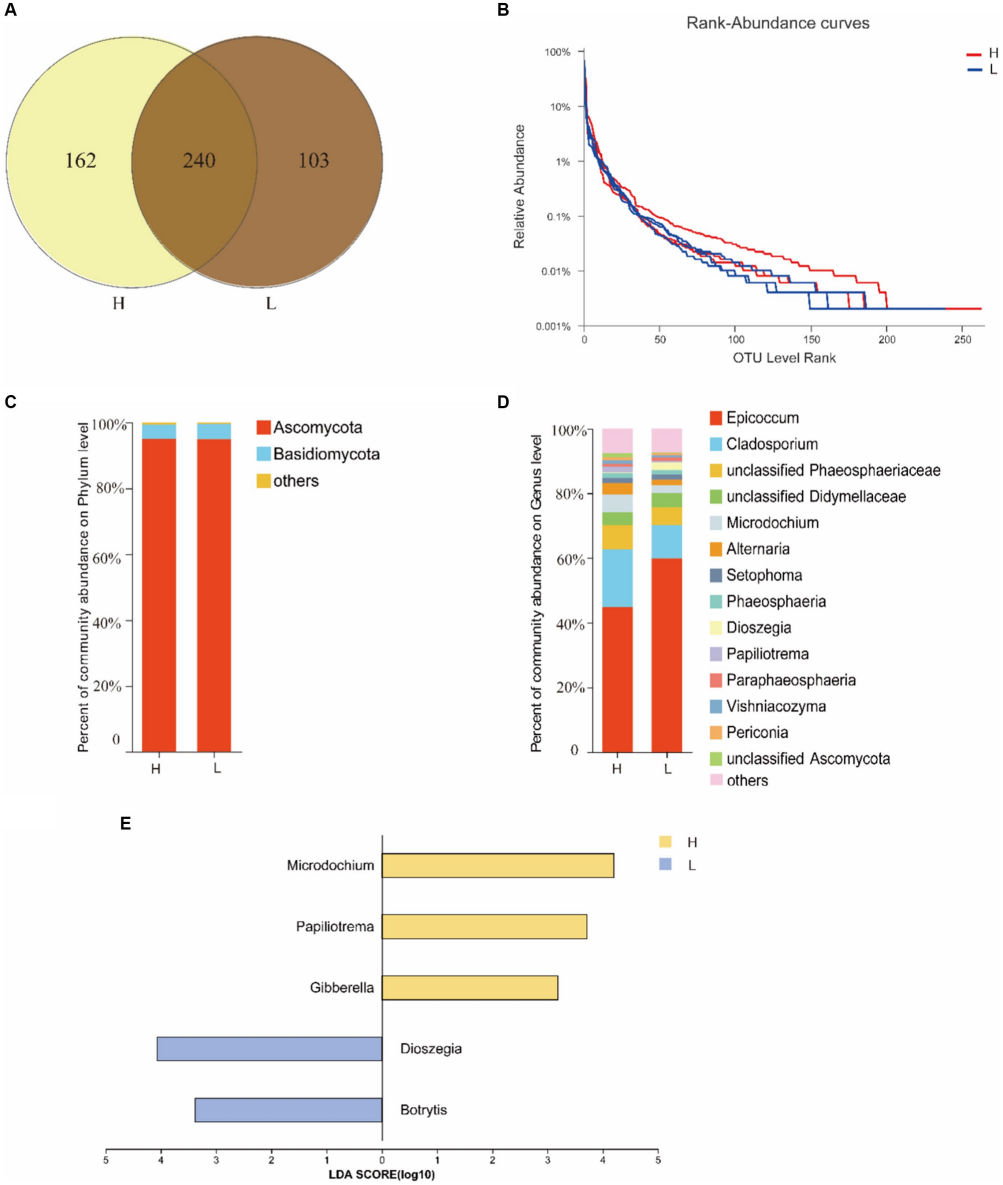
The diversity of seed fungal endophytes of *I. cylindrica* from different heavy metal-contaminated environments. **(A)** The Venn diagram analysis of OTUs. **(B)** The rank curve based on OTU abundance. **(C,D)** Relative abundance of seed endophytic fungal at phylum and genus level. **(E)** The indicator species of seed endophytic fungal at genus level. H, Heavy metal heavily contaminated site; L, Heavy metal lightly contaminated site.

The OTUs of endophytic fungal in seeds were more abundant at site H (402) than at site L (343), and 240 OTUs were shared by both two sites (Figure 2A). Rank-Abundance curve indicated that the richness of seed endophytic fungi at site H was relatively higher than that at site L (Figure 2B).

The results of ITS sequencing of seed endophytic fungal were assigned to 6 phyla, 323 classes, 56 orders, 123 families, and 222 genera. Ascomycota was the most dominant fungal phylum (Figure 2C). At genus level, *Epicoccum* sp. displayed the most relative abundance in both sites H and L, with 44.78 and 55.91%, respectively. Other dominant genera in site H followed by *Cladosporium* sp. (17.87%) and unclassified Phaeosphaeriaceae (7.57%), with higher relative abundance than site L (Figure 2D), but neither of them was a indicator species at site H at genus level (Figure 2E). According to α-diversity analysis, it was found that *I. cylindrica* seed endophytic fungal diversity and richness of site H were higher than site L, but the difference was not significant (Table 2).

#### 3.1.3 The effect of environmental factors on seed endophyte community

**Figure 3:**
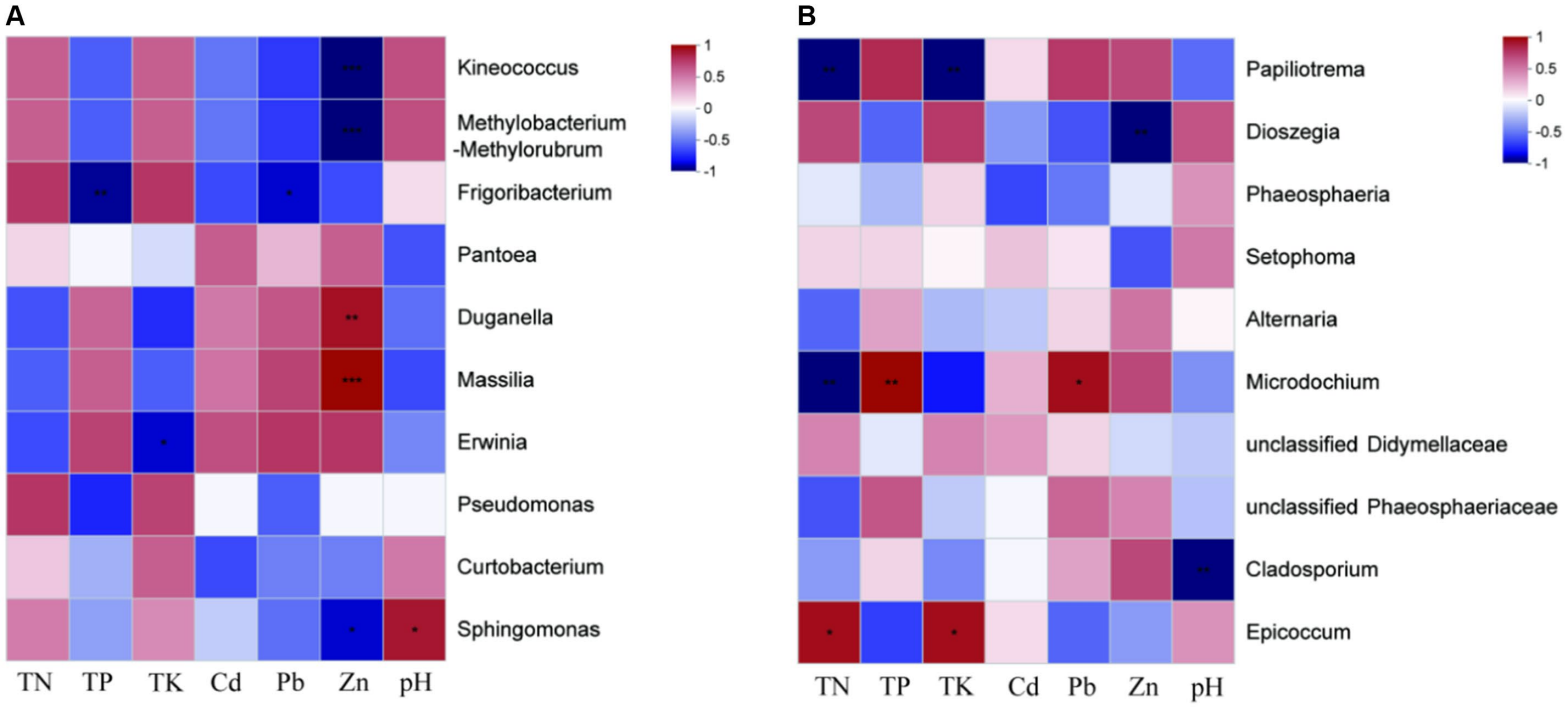
Spearman correlation heatmap of *I. cylindrica* seed endophytes at genus level. **(A)** Seed endophytic bacteria. **(B)** Seed endophytic fungi. “*” indicates statistically significant difference (*p < 0.05; **p < 0.01; ***p < 0.001).

The contents of HMs (Cd, Pb, Zn) at site H were significantly higher than those at site L, while the contents of nutrients (TN and TK) at site L were significantly higher than those at site H (Table 1). *Massilia* sp. had the highest relative abundance of seed endophytic bacteria of *I. cylindrica* in site H. Spearman’s rank correlation coefficient analysis showed a significant positive correlation between *Massilia* sp. and Zn (Figure 3A). In addition, the result showed that *Cladosporium* sp. was negatively correlated with pH. Similarly, *Microdochium* sp. was a relatively dominant fungus at site H, and its abundance was significantly different from that at site L. Spearman’s rank correlation coefficient analysis indicated that *Microdochium* sp. was significantly positively correlated with TP and Pb (Figure 3B).

### 3.2 The effect of HM-contamination on soil microbial diversity and community structure

#### 3.2.1 Microbial diversity of bare soils and rhizosphere soils of *Imperata cylindrica* in HM heavily contaminated site

A total of 263,311 and 355,189 sequences high-quality of bacteria and fungi were obtained after demultiplexing and filtration steps. The mean number of valid bacterial sequences was 31,563, whereas the mean number of valid fungal sequences was 32,254. These sequences were divided into 2,434 and 1,933 different OTUs, respectively, with 97% similarity.

**Figure 4:**
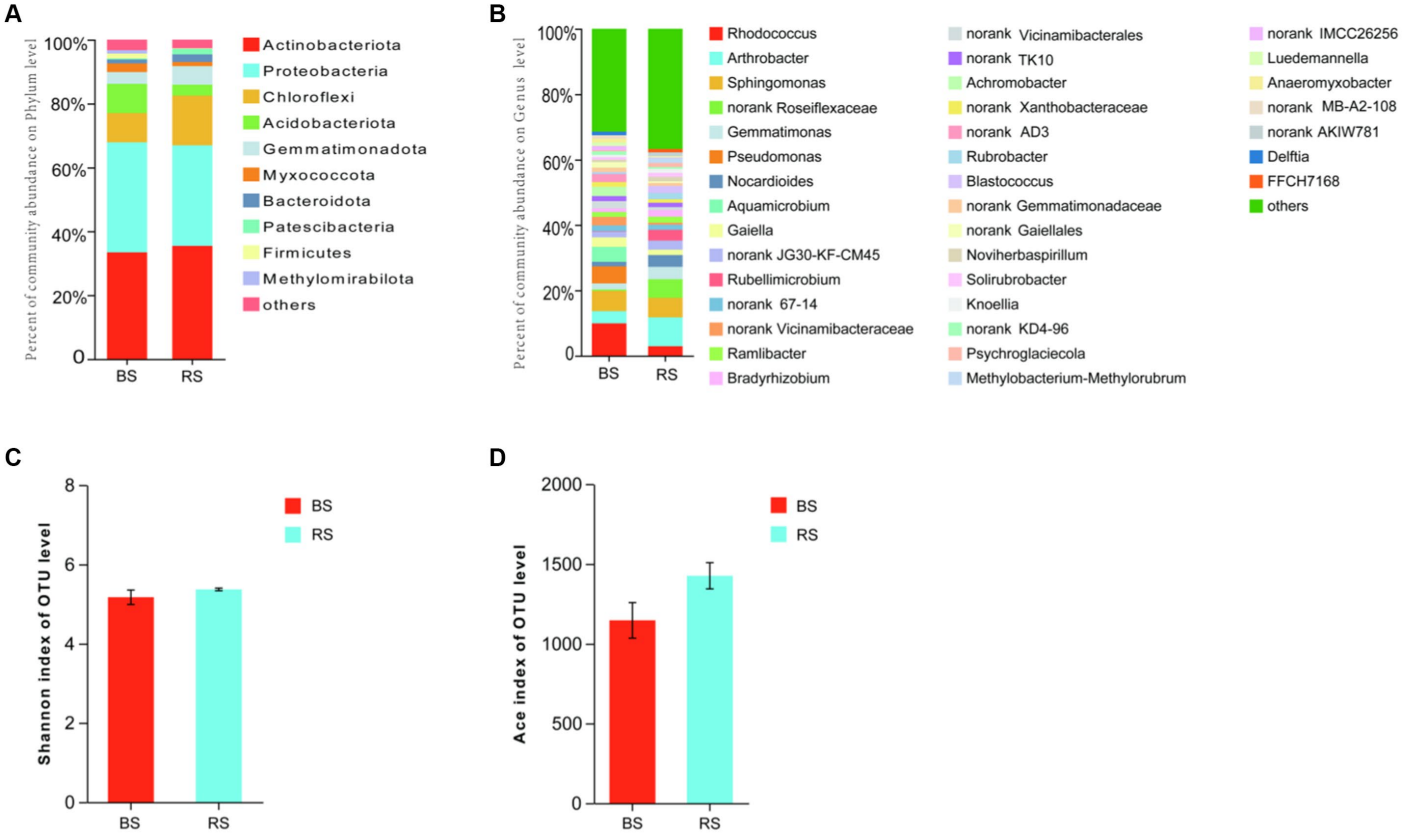
Bacterial diversity in bare soils and rhizosphere soils in HM heavily contaminated site. **(A,B)** Relative abundance of soil bacterial composition at the level of phylum and genus. **(C)** Shannon’s H′. **(D)** Ace index. BS, Bare soils of site H; RS, Rhizosphere soils of site H.

The results of 16S rRNA sequencing of soil bacterial were assigned to 32 phyla, 91 classes, 312 orders, 316 families, and 570 genera. Actinobacteriota displayed the most relative abundance in both soil samples (Figure 4A). At genus level, *Rhodococcus* sp. was the most dominant genus in BS (Bare soil) (9.90%). Contrary to this, the most dominant genus of RS (Rhizosphere soil) was *Antrobacter* sp. (8.99%). Many OTUs were unclassified at genus level in both soil samples (Figure 4B). The results of α-diversity (Shannon’s H′ and Ace indexes) analysis indicated that BS and RS was not significantly different (Figures 4C,D).

**Figure 5:**
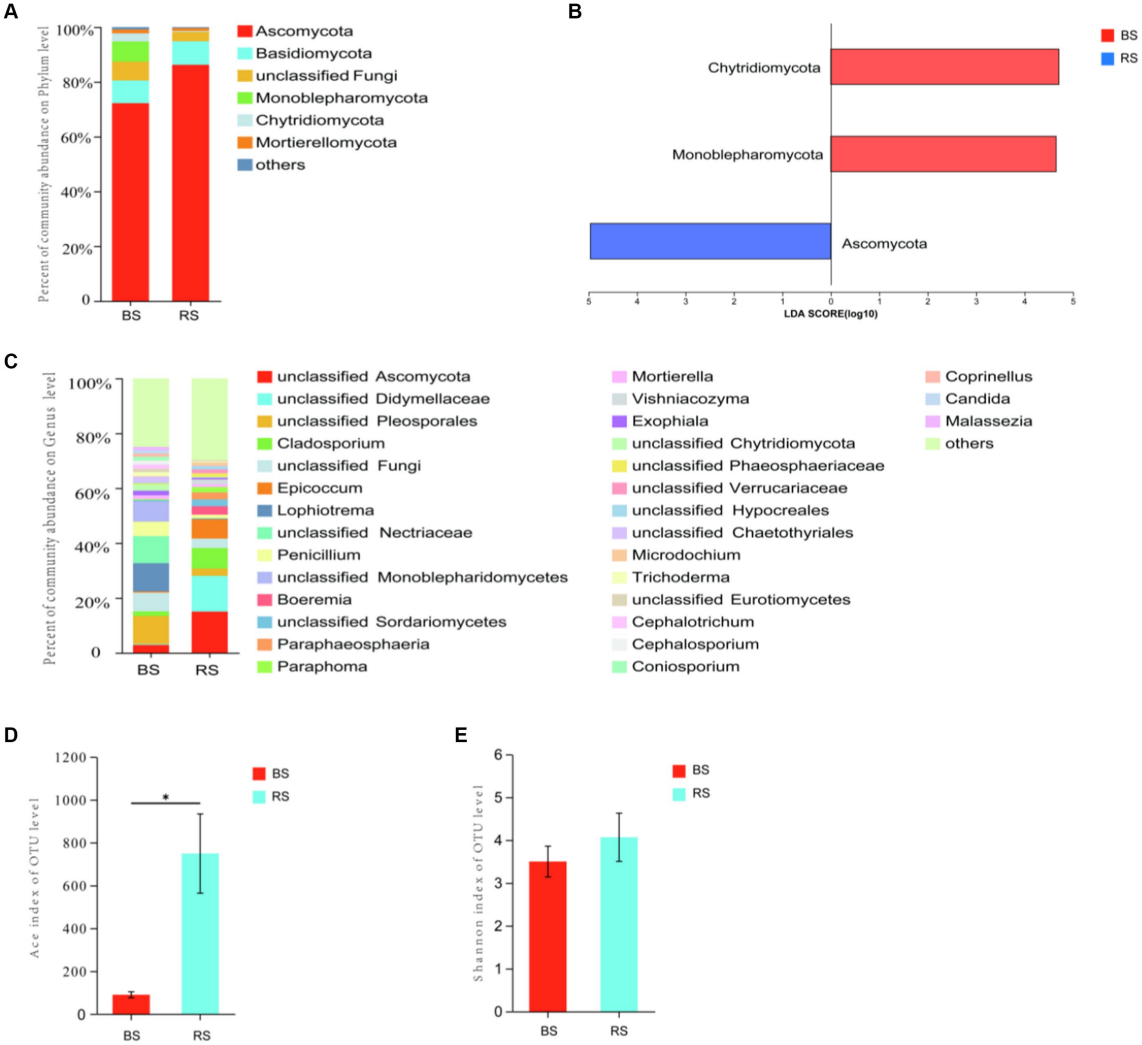
Fungal diversity in bare soils and rhizosphere soils in HM heavily contaminated site. **(A)** Relative abundance of soil fungal composition at the level of phylum. **(B)** The indicator species of soil fungal at phylum level. **(C)** Relative abundance of soil fungal composition at the level of genus. **(D)** Ace index. **(E)** Shannon’s H′. “*” indicates statistically significant difference (*p < 0.05, *t*-test). BS, Bare soils of site H; RS, Rhizosphere soils of site H.

The results of ITS sequencing of soil fungal were assigned to 9 phyla, 37 classes, 98 orders, 233 families, and 497 genera. Ascomycota displayed the most relative abundance in both soil samples (Figure 5A), and the relative abundance of RS was significantly higher than that of BS (*p* = 0.0495, Kruskal-Wallis sum-rank test) (Figure 5B). At the same time, Ascomycota is also the indicator species of soil fungal at phylum level of RS (Figure 5B). At genus level, unclassified Ascomycota and unclassified Didymellaceae were the most dominant fungi in BS (15.03%) and in RS (10.28%) (Figure 5C). The results of Ace index indicating that the species richness of RS was significantly higher than that of BS (*p* = 0.0237, *t*-test) (Figure 5D), while the results of Shannon index showed that the diversity of the two soil samples was not significantly different (Figure 5E).

#### 3.2.2 The effect of environmental factors on bare soils and rhizosphere soils microbiome of *Imperata cylindrica* in HM heavily contaminated site

**Figure 6:**
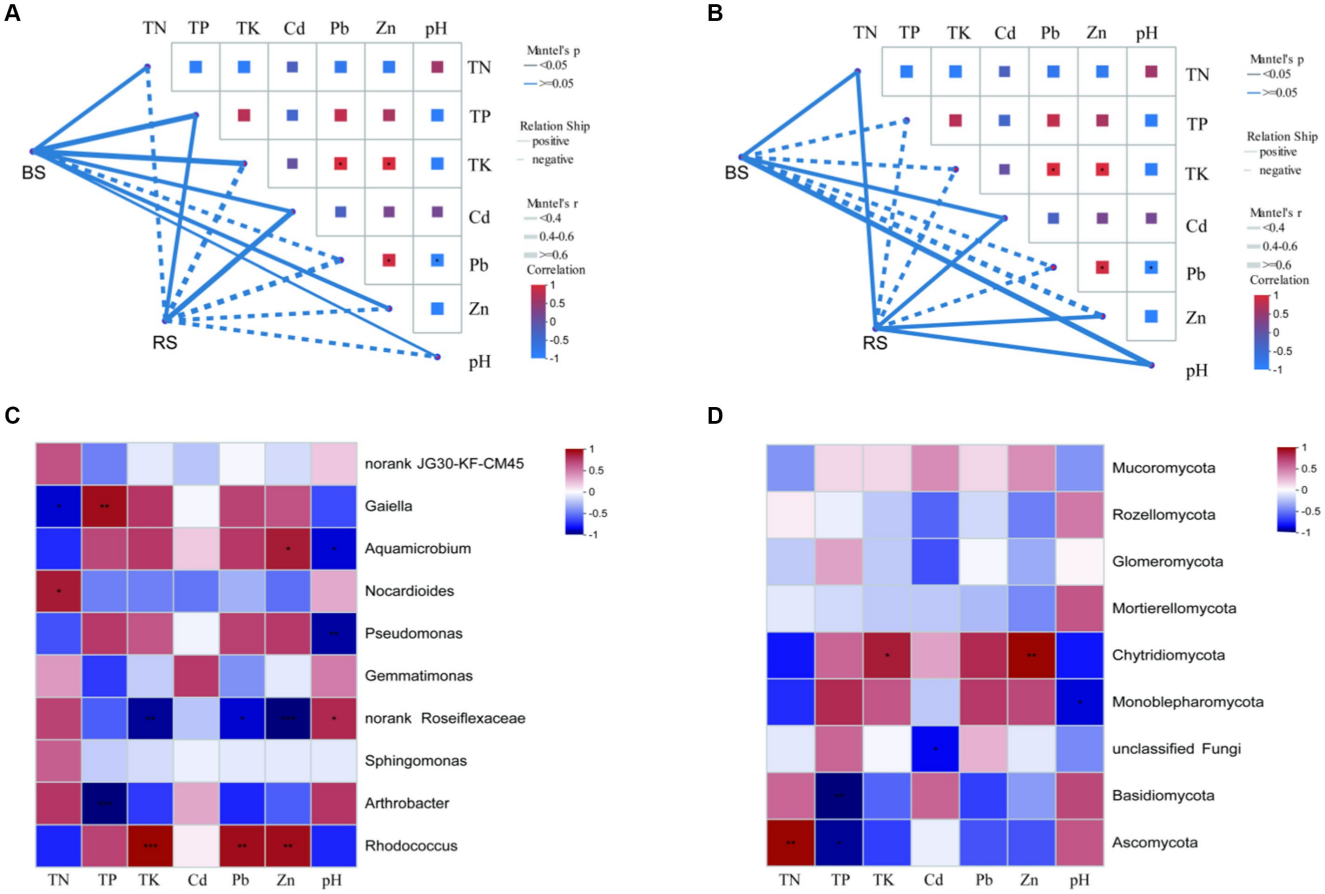
Heat map of bacterial and fungal correlation with environmental factors in bare soils and rhizosphere soils of HM heavily contaminated site. **(A,B)** Mantel-test Network heatmap of bacteria and fungi at phylum level. **(C)** Spearman’s rank correlation coefficient heatmap of bacteria at genus level. **(D)** Spearman’s rank correlation coefficient heatmap of fungi at phylum level. “*” indicates statistically significant difference (*p < 0.05; **p < 0.01). BS, Bare soils of site H; RS, Rhizosphere soils of site H.

The contents of HMs (Pb and Zn) and nutrients (TP and TK) of BS were significantly higher than that of RS (Table 1). Mantel-test Network heatmap showed that at the phylum level, bacterial communities in BS were negatively correlated with Pb, while bacterial communities in RS were only positively correlated with TP and Cd, but the differences were not significant (Figure 6A). For fungi, at phylum level, Mantel-test Network heatmap shown that fungal communities in BS were positively correlated with TN, Cd, and pH, while fungal communities in RS were positively correlated with TN, Cd, Zn, and pH, but the differences were not significant (Figure 6B). Spearman’s rank correlation coefficient analysis showed that *Rhodococcus* sp. was significantly positively correlated with Pb, Zn, and TK, and *Arthrobacter* sp. was significantly negatively correlated with TP at genus level (Figure 6C). For fungi, Spearman’s rank correlation coefficient analysis indicated that Ascomycota was significantly positively correlated with TN, but significantly negatively correlated with TP (Figure 6D).

## 4 Discussion

Previous researches has demonstrated that *I. cylindrica* plays an important role in plant community restoration in the mining district (Zheng et al., 2019; Jia et al., 2020). Meanwhile, various research indicated that plant-associated microorganisms can enhance host plants’ stress tolerance (Trivedi et al., 2020). However, the microbiome of pioneer plants growing in the bare mine tailing with higher concentrations of HMs as well as its role are still unknown. The present study indicated that HM contamination reduced the endophytic bacterial richness of *I. cylindrica* seeds. Proteobacteria was the most dominant of *I. cylindrica* seeds in site H. Some studies have established that Proteobacteria is relatively more abundant in plants growing in HM-contamination environments, as it is composed of many facultative anaerobic members that can survive in extreme pH environments, proving to be taxa most tolerant to HMs (Sun et al., 2010; Sánchez-López et al., 2018; Kasemodel et al., 2019). Simultaneously, some Proteobacteria are plant growth-promoting bacteria that have been proven to fix nitrogen symbiotically with host plants (Wu et al., 2022). *Massilia* sp. is a genus of Proteobacteria. It was found to be a dominant genus in site H, and its relative abundance was significantly higher than that in site L. At the same time, it is an indicator species for seed endophytic bacterial in site H. Moreover, Spearman’s rank correlation coefficient analysis demonstrated that *Massilia* sp. had a significant positive correlation with Zn concentration in soils. Wang et al. (2021) found that *Massilia* was a potential Zinc-mobilizing species in soil and that it was significantly positively correlated with Zn concentration in wheat grains. Analogously, Wang et al. (2022) found that *Massilia* sp. had a significant positive relationship with DTPA-Pb (diethylene-triamine-pentaacetic-acid-Pb) in soils and Pb accumulation in roots. *Massilia* sp. was isolated from sludge, farmland, and mining soils contaminated by heavy metals, suggesting that *Massilia* sp. is an important microorganism in HM-contaminated soils (Bensidhoum and Nabti, 2019). In addition, it has also been reported as *Massilia* sp. with strong phosphate solubilizing ability (Zheng et al., 2017). In terms of fungi, the diversity and richness of seed endophytes of *I. cylindrica* in site H were higher than those in site L, but the difference was not significant. The results indicated that HM contamination had no significant effect on seed endophytic fungal diversity and richness. However, relative abundance of *Microdochium* sp. in site H was significantly higher than that in site L. At the same time, it is an indicator species for endophytic fungi in seeds in site H. It was found that *Microdochium* sp. could promote the growth of *Hordeum vulgare* L. under Cd stress, and significantly increase Cd accumulation in barley roots (Shadmani et al., 2021). In addition, it has been reported that *Microdochium* sp. is well tolerant to Pb and it has also been found that the accumulation of Pb in *Microdochium* sp. increases with the concentration of Pb in the growth media (Parada et al., 2022). Similarly, our earlier study found that *Microdochium* sp. was tolerant to 2000 mg/L Pb. This was consistent with the results of correlation analysis of soil environmental factors, which showed a significant positive correlation between *Microdochium* sp. and Pb, indicating that its large presence in the seeds of *I. cylindrica* in the tailing area may improve the host’s Pb-tolerance. At the same time, a previous study found that *Microdochium* sp. is capable of synthesizing IAA *in vitro* (Rothen et al., 2018). Based on the above results, we supposed that *Massilia* sp. and *Microdochium* sp. may play an important role in the tailing area plant community restoration. For this reason, a series of pot experiments used to investigate the effects of *Massilia* sp. and *Microdochium* sp. on seed germination, plant growth, and rhizosphere microbiome reshaping of *I. cylindrica* is already in progress.

It is well known that in addition to endophytes, rhizosphere microorganisms also play an important role in the process of vegetation community restoration in the tailing area (Wang et al., 2023). Plant growth has been reported to alter the abundance of specific functional microorganisms (Sun et al., 2018; Zhu et al., 2023). In the present study, it was found that the microbial diversity and richness in rhizosphere soils of *I. cylindrica* were higher than those in bare soils, and there was a significant difference in fungal richness between the two soil samples. Ascomycota displayed the most relative abundance in rhizosphere soils, and it was significantly higher than that in bare soils. The same phenomenon was observed in other studies (Bourceret et al., 2016; Rosatto et al., 2019). We suggested that one of the reason for the higher relative abundance of Ascomycota in rhizosphere soils of *I. cylindrica* may be its ability to efflux complex spores providing extra-resistant against the toxicities of HMs (López-González et al., 2015; Liu et al., 2022). Simultaneously, Spearman correlation analysis showed that Ascomycota was positively correlated with total nitrogen content. Regarding bacteria, *Arthrobacter* sp. is the most dominant genus in rhizosphere soils, and its relative abundance in rhizosphere soils was significantly higher than that in bare soils. It has been demonstrated that *Arthrobacter* sp. not only contained HM-resistance genes but also strongly correlated with higher siderophore and IAA production (Rosatto et al., 2019; Senthil Kumar et al., 2023). Meanwhile, Wan et al. (2020) found that *Arthrobacter* sp. could utilize multiple phosphorus sources. Therefore, we suggested that to adapt HM-contamination environment, *I. cylindrica* recruits many beneficial microorganisms to colonize in the rhizosphere, which helps it enhance HM resistance and nutrient uptake.

Some studies have shown that endophytes can help host plants resist stressful environments by regulating root exudates and remodeling rhizosphere microbiome structure (Li et al., 2019; Wang et al., 2023). Jin et al. (2022) found that under Cd stress, inoculation of *Epichloe gansuensis* could increase the contents of organic acids and amino acids in root exudates of *Achnatherum inebrians*, thereby recruiting different rhizosphere microbe and enhancing the host plant resistance to Cd stress. We suggested that the endophytic consortium in the seeds of *I. cylindrica* in the HM heavily contaminated site might increase the relative abundance of microorganisms in rhizosphere soils related to HM-fixation and soil nutrient cycling by regulating root exudates during plant growth, which would help plants adapt to HM stress (Liu et al., 2022). However, the functional mechanisms by which the interactions among plants, endophytes, and soils enhance the “site fitness” of host plants remain unclear. Studying the combined effects of endophytes and rhizosphere soil microorganisms can better reveal the reasons for the restoration of natural vegetation in the tailing area.

## 5 Conclusion

HM-contamination significantly reduced the richness of endophytic bacteria in seeds, but increased the abundance of resistant species. The colonization of *I. cylindrica* significantly increased the richness of fungi in rhizosphere soils. Ascomycota was positively correlated with the total nitrogen, and it may enhance the nitrogen fixation of *I. cylindrica*, thus promoting its growth in extreme environment. The survival of *I. cylindrica* in HM severely contaminated environment may mainly be through recruiting more microorganisms that can enhance its nutrition supply. The study indicated that the seed endophytic community of *I. cylindrica* from HM heavily contaminated site differed from that of uncontaminated site, therefore, the future work will focus on discovering the function and mechanism of the special endophytes of pioneer plants from HM-contaminated site, and further exploring them in the tailing area restoration.

## Data availability statement

The datasets presented in this study can be found in online repositories. The names of the repository/repositories and accession number(s) can be found at: https://www.ncbi.nlm.nih.gov/, PRJNA1037316.

## Author contributions

WM: Conceptualization, Methodology, Writing – original draft, Writing – review & editing. YW: Conceptualization, Methodology, Writing – original draft, Writing – review & editing. QL: Formal analysis, Investigation, Writing – review & editing. YX: Formal analysis, Investigation, Writing – review & editing. WT: Formal analysis, Investigation, Supervision, Writing – review & editing. HH: Supervision, Writing – review & editing. XJ: Supervision, Writing – review & editing. HL: Conceptualization, Funding acquisition, Methodology, Writing – review & editing, Project administration, Resources.
